# Impact of Postcalving Intrauterine Enzymes Therapy on Uterine Health, Fertility, Biochemical Indices, and Milk Production in Dystocia‐Affected Buffaloes

**DOI:** 10.1155/vmi/5748390

**Published:** 2026-05-12

**Authors:** Nakul Gulia, Mrigank Honparkhe, Amarjeet Bisla, Ashwani K. Singh, Prahlad Singh

**Affiliations:** ^1^ Department of Veterinary Gynaecology and Obstetrics, Guru Angad Dev Veterinary and Animal Sciences University, Ludhiana, 141004, Punjab, India, gadvasu.in

**Keywords:** blood lactate, buffalo, creatine kinase, dystocia, proteolytic enzymes, uterine health, uterine involution

## Abstract

This study investigated the effect of postcalving intrauterine proteolytic enzyme therapy on uterine involution, ovarian rebound, milk yield, biochemical indices, and postpartum fertility in dystocia‐affected buffaloes. Thirty buffaloes were randomly assigned to two groups. The treatment group (Group I; *n* = 15) received a combination of proteolytic enzymes, that is, trypsin (16 mg), chymotrypsin (16 mg), and papain (8 mg) dissolved in 500 mL normal saline (NS) intrauterine after relief of dystocia, while the control group (Group II; *n* = 15) received 500 mL NS as a placebo. Average uterine involution scores on Day 20, Day 45, and combined were significantly better (*p* < 0.05) in treated buffaloes, with 73.33% of controls exhibiting delayed involution. Treated animals showed a significant reduction (*p* < 0.05) in uterine infections. Blood lactate (bLac) and creatine kinase (CK) levels were lower (*p* < 0.05) in the treatment group on both days, with bLac showing a strong negative correlation (*p* < 0.05) and CK a moderate negative correlation with uterine involution scores. Milk production was significantly higher (*p* < 0.05) in the treatment group on Day 20 and Day 45. The cumulative pregnancy rate by Day 250 postpartum was greater in treated buffaloes (80.0%) than controls (46.67%), with a tendency toward improvement (*X* ^2^ = 3.35; *p* = 0.067). In conclusion, intrauterine administration of proteolytic enzymes immediately after assisted delivery accelerated uterine involution, reduced uterine infections, improved milk yield, and enhanced fertility in dystocia‐affected buffaloes. Additionally, bLac and CK were identified as potential biomarkers for monitoring uterine health in the postpartum period.

## 1. Introduction

The profitability of dairy enterprises largely depends on the number of calf crops produced per female, which is directly influenced by the inter‐calving interval. In water buffaloes (*Bubalus bubalis*), achieving the target of one calf every 13–14 months requires limiting open days to ≤ 120. Postpartum fertility depends on a healthy peripartum period, characterized by normal parturition, timely uterine involution, and resumption of ovarian cyclicity [1, 2]. Any disturbance during this phase prolongs the calving interval and reduces both lifetime reproductive and productive efficiency.

Dystocia, a common obstetrical problem in dairy buffaloes, adversely affects the puerperal period by predisposing animals to uterine infections, extending the open days interval, and lengthening the inter‐calving period, ultimately reducing the lifetime number of calves per female [3, 4]. The incidence of bacterial infection following dystocia can be as high as 89.9%, compared to 63.3% in normally calved animals, resulting in delayed uterine involution [5]. Uterine involution, the return of the uterus to its prepregnant size and function, depends on factors such as myometrial contractions, endometrial regeneration, and bacterial clearance [6, 7].

Delayed resumption of estrus in dystocia‐affected buffaloes is linked to endotoxins, such as lipopolysaccharide (LPS), released by invading bacteria. These endotoxins suppress hypothalamic GnRH secretion, inhibit pulsatile LH release, and reduce pituitary responsiveness to GnRH. In addition, calving compromises the cervix, vagina, and vulva, allowing opportunistic pathogens to colonize the uterine lumen, leading to inflammation and endometrial lesions that delay involution and impair embryo survival [8–10].

Hormonal therapies like oxytocin, prostaglandin F_2_ *α* (PGF_2_ *α*), and GnRH have been tested in dystocia‐affected buffaloes to accelerate uterine involution and cyclicity, but behavioral estrus still often appears only after ∼4 months [11, 12]. Moreover, early postpartum use of oxytocin or PGF_2_ *α* shows no significant benefit in involution rate [13].

Proteolytic enzymes such as chymotrypsin, trypsin, and papain possess fibrinolytic, proteolytic, and immunomodulatory properties, aiding cellular defense, breakdown of necrotic tissue, and stimulation of smooth muscle contractility, potentially supporting uterine involution [14–16]. Intrauterine enzyme therapy has been successfully used to treat endometritis in cattle [17] and buffaloes [18], as well as repeat breeder cases [19–21]. However, studies on their effect specifically in dystocia‐affected buffaloes, particularly regarding uterine involution, cyclicity, fertility, and milk yield, are lacking.

The extent of uterine injury after dystocia can be assessed through biochemical markers such as blood lactate (bLac), aspartate aminotransferase (AST), and creatine kinase (CK) [22]. Elevated bLac reflects anaerobic metabolism due to muscular strain and ischemia and is reported in uterine torsion cases [23]. Increased CK and AST levels indicate muscle damage and inflammation [24–26]. Despite their diagnostic potential, few studies have investigated these markers in relation to dystocia, and virtually none have examined their dynamics following proteolytic enzyme therapy in buffaloes.

Given the limitations of hormonal therapy and the promising attributes of proteolytic enzymes, it is hypothesized that intrauterine administration of a proteolytic enzyme combination immediately after assisted delivery would optimize uterine involution, uterine health, milk production, and postpartum fertility in dystocia‐affected buffaloes.

## 2. Materials and Methods

The present study was conducted on dystocia‐affected buffaloes presented at the University Clinics, Guru Angad Dev Veterinary and Animal Sciences University (GADVASU), Ludhiana, Punjab, India, located between 30°34′–31°01′ N latitude and 75°18′–76°20′ E longitude. A total of 30 buffaloes suffering from dystocia with a good prognosis were enrolled. Obstetrical interventions were performed according to the etiology of dystocia (provided as supporting data). Ethical approval was obtained from the Committee for the Purpose of Control and Supervision of Experiments on Animals (CPCSEA) [Letter No. V‐11011 (13)/2/2021‐CPCSEA‐DADF].

**FIGURE 1:**
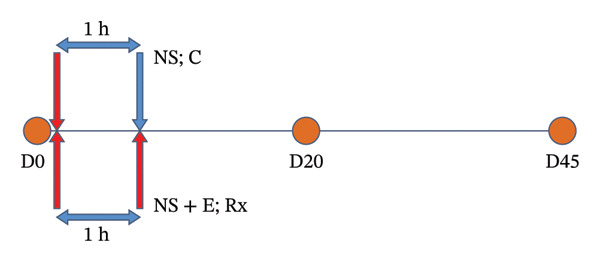
Experimental design of the study depicting administration of intrauterine proteolytic enzymes (trypsin 16 mg, chymotrypsin 16 mg, and papain 8 mg dissolved in 500 mL normal saline) to treatment group animals while only a placebo of 500 mL normal saline is given to control group animals after 1 h of assisted delivery. The animals were further examined on Days 20 and 45 postpartum (D0; day of handling of dystocia; D20 and D45: 20 and 45 days after handling of dystocia; NS: normal saline; C: control group; E: enzyme therapy; Rx: treatment group).

The experimental design is depicted in Figure 1. Buffaloes were randomly allocated to two groups: Group I (treatment; *n* = 15) and Group II (control; *n* = 15). After thorough anamnesis and per‐vaginal examination, the cause of dystocia was diagnosed and corrected using appropriate obstetrical procedures such as rolling (for uterine torsion), mutation, or fetotomy. Both groups were balanced for cause and type of dystocia (Supporting Tables 1 and 2).

Within 1 hour of assisted delivery, Group I animals received an intrauterine formulation of proteolytic enzymes, namely, trypsin (16 mg; Sigma‐Aldrich, Product Code 048K7021), chymotrypsin (16 mg; Sigma‐Aldrich, Product Code 086K7695), and papain (8 mg; Sigma‐Aldrich, Product Code 76220) dissolved in 500 mL normal saline (NS). The dosage was double that used for subclinical endometritis [18] and diluted in a larger NS volume, considering the size of the gravid uterus. Group II animals received a placebo of 500 mL NS. This higher dose of the enzymatic cocktail was selected in the current study to account for the larger uterine volume and the increased amount of inflammatory exudate, necrotic tissue, and fibrin debris present in the immediate postpartum uterus following dystocia, keeping in view that it may require greater enzymatic activity for effective tissue debridement and uterine clearance.

Before enzyme or placebo administration, both groups received conventional postpartum therapy, including intravenous fluids, antimicrobials, antioxidants, nonsteroidal anti‐inflammatory drugs (NSAIDs), multivitamins, ecbolics, liver extract, and rumenotorics (Supporting Table 3).

Rectal palpation and trans‐rectal ultrasonography (Easi‐Scan, IMV Imaging, USA) were performed on Days 0, 20, and 45 to assess genital location, uterine horn/body size, and uterine infections. Uterine involution scores were calculated as per Gulia et al. [27], which included rectal palpation along with ultrasonographic parameters, including diameters of the cervix, uterine body, gravid and nongravid uterine horns, and presence of types of uterine fluid along with location of genitalia in the pelvic cavity. Four different scores (1–4) were given to each parameter on Days 20 and 45 postpartum. Based on the total score obtained (out of 24 maximums on each day) and their sum (Days 20 and 45 postpartum scores), three different uterine involution gradings (early, normal, and delayed) were given for the extent of uterine involution. The scoring system was previously developed and validated in dystocia‐affected buffaloes using sequential clinical and ultrasonography observations, demonstrating good agreement between observers and consistency in assessing uterine recovery.

The animals under both groups were assessed for the presence of uterine infections, that is, puerperal metritis, clinical endometritis, and subclinical endometritis as per Sheldon et al. [28]. The puerperal metritis and clinical endometritis were diagnosed by the observation of the clinical picture, while, subclinical endometritis was diagnosed by uterine cytology taken by cytobrush and staining with Giemsa stain. The buffaloes having more than 7% polymorphonuclear cell count (PMNs) on cytology examination were classified as suffering from subclinical endometritis [18].

All animals were also subjected to blood sampling on the day of dystocia (Day 0), Day 20, and Day 45 postpartum, and 10 mL of blood was collected in serum vials. The serum was separated and subjected to analysis of blood biochemical parameters within 3 h of the sampling. The serum biochemical parameters assessed were bLac concentration, AST, creatine phosphokinase/kinase (CK), total proteins (TP), blood urea nitrogen (BUN), and serum creatinine. These parameters were assessed using analytical kits as per instructions provided by the manufacturers (Ortho Clinical Diagnostics). The automatic biochemical analyzer (Vitro 350 system) was used for estimation of these parameters.

Reproductive performance parameters included spontaneous estrus rate (%), interval from calving to first estrus (days), calving to conception interval (days), and pregnancy rate (%) calculated until 120 days, between 120 and 250 days, and cumulatively until 250 days postpartum. The spontaneous estrus rate (%) was calculated by dividing the number of animals that showed spontaneous estrus by the total number of animals in that group and multiplying by 100. The interval from calving to first postpartum estrus (days) and calving to conception interval (interval from calving to successful conception; days) were also calculated for each animal, and then the average was taken for the group. The overall pregnancy rate (number of animals pregnant/total number of animals, multiplied by 100; %) was calculated till Day 120 postpartum, after 120–250 days postpartum, and overall till Day 250 postpartum.

### 2.1. Statistical Analysis

Data were analyzed using GraphPad Prism v9.0.0 (121). Results are expressed as mean ± SEM (standard error of mean) or percentages, with significance set at *p* < 0.05 and trends toward effect of therapy at *p* < 0.10. The chi‐square test compared pregnancy rates; Student′s *t*‐test compared groups, and repeated measures one‐way ANOVA evaluated changes over time. Pearson’s correlation coefficients (*r*) were calculated between uterine involution scores and biochemical, ultrasonographic, and milk production parameters. Descriptive statistics were used to evaluate enzyme therapy effects. Binary logistic regression assessed the combined influence of significant variables on pregnancy outcome, using the model as follows:

(1) Logit p=logp1−p=β0+βixi,

where logit (*p*) represents log odds of pregnancy, *β*_0_ represents a constant, *β*_*i*_ represents parameters associated with factors, and x_i_ represents fixed factors related to the study, which included enzyme given, uterine infection at Day 20, uterine infection at Day 45, and uterine involution score at Day 45. Further, exp(β), that is, odds ratio, was obtained for each fixed factor.

## 3. Results

**FIGURE 2:**
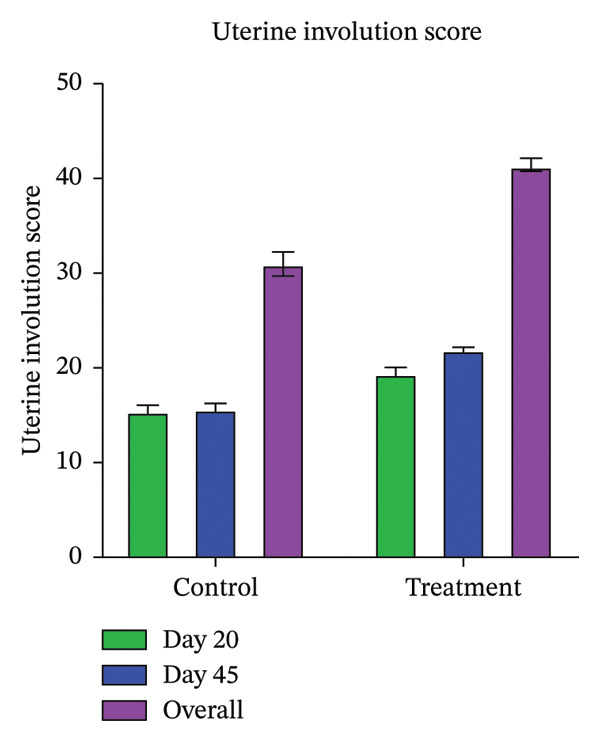
The bar diagram depicting the uterine involution scorecard on Days 20, 45 and on both Days 20 and 45 postpartum in the control and treatment groups.

**FIGURE 3:**
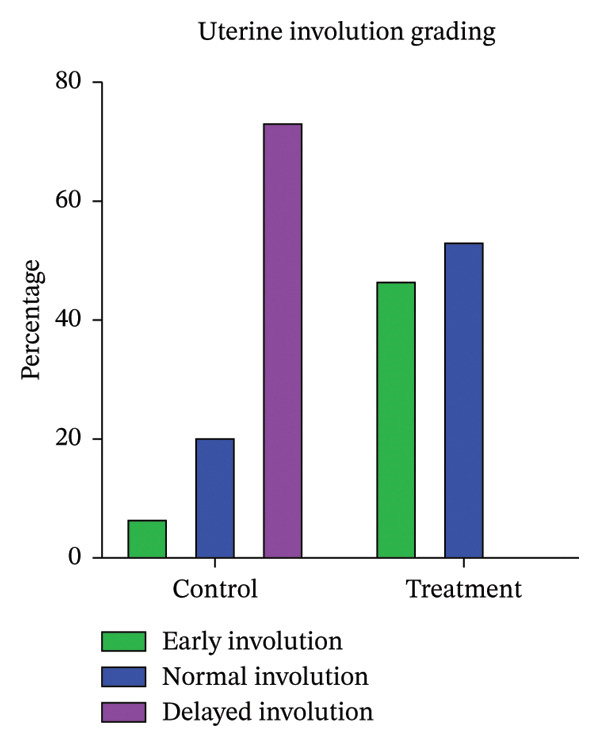
The bar diagram depicting uterine involution grading in early, normal, and delayed uterine involution in control and treatment groups.

The sonographic assessment of uterine involution (Table 1) revealed significantly higher (*p* < 0.05) average uterine involution scores on Days 20 and 45, as well as in the combined score, in the treatment group compared with controls. Within the treatment group, the score was greater (*p* < 0.05) on Day 45 than on Day 20, while no significant difference (*p* > 0.05) was observed in controls (Figure 2). Delayed uterine involution occurred exclusively in the control group (73.33%), with no such cases in treated animals (Figure 3). The incidence of puerperal metritis/clinical endometritis up to Days 20 and 45 was significantly lower (*p* = 0.007) in treated buffaloes (Table 2), and subclinical endometritis was reduced (*p* = 0.008; Table 3).

**TABLE 1:** Ultrasonographic biometry of cervix and uterus following different treatments in dystocia‐affected buffaloes.

S. No.	Parameter	Control (*n* = 15)		Treatment (*n* = 15)	
		Day 20	Day 45	Day 20	Day 45
1.	Cervix diameter (mm)	34.81 ± 1.51^a^	29.04 ± 1.15^b^	28.50 ± 1.01^b^	22.63 ± 0.74^c^
2.	Uterine body diameter (mm)	46.19 ± 1.05^a^	38.27 ± 1.00^b^	38.63 ± 1.08^b^	30.05 ± 0.82^c^
3.	Gravid uterine horn diameter (mm)	33.65 ± 0.88^a^	28.12 ± 0.80^b^	28.90 ± 0.84^b^	20.80 ± 0.55^c^
4.	Nongravid uterine horn diameter (mm)	29.81 ± 0.92^a^	25.00 ± 1.02^b^	25.64 ± 0.74^b^	18.15 ± 0.47^c^

*Note:* Superscripts a, b, c represent significant different values within rows (*p* < 0.05).

**TABLE 2:** Percentage of animals developing uterine infections in different groups.

Type of uterine infections	Control (*n* = 15)	Treatment (*n* = 15)	*P*‐value
Puerperal metritis/clinical endometritis till Day 20 postpartum (%)	73.33^a^ (11/15)	26.67^b^ (4/15)	0.007
Subclinical endometritis on Day 45 postpartum (%)	53.33^∗^ (8/15)	6.67^#^ (1/15)	0.008

*Note:* Superscripts a, b represent significant different values within rows (*p* = 0.007). Superscripts ^#^, ^∗^ represent significant different values within rows (*p* = 0.008).

**TABLE 3:** Mean ± SEM number of different‐sized ovarian follicles on Days 20 and 45 postpartum in dystocia‐affected buffaloes following treatment.

S. No.	Ovary	Parameter	Control (*n* = 15)		Treatment (*n* = 15)	
			Day 20	Day 45	Day 20	Day 45
1.	Left	≤ 6 mm	3.5 ± 0.53^A^	2.6 ± 0.21	3.7 ± 0.33^aA^	2.7 ± 0.32^b^
		6.01–7.99 mm	0.38 ± 0.14	0.15 ± 0.10	0.25 ± 0.10	0.05 ± 0.05
		≥ 8.00 mm	0.00 ± 0.00	0.69 ± 0.13	0.25 ± 0.10	0.55 ± 0.11
		Overall	3.92 ± 0.51	3.46 ± 0.24	4.20 ± 0.27^A^	3.30 ± 0.33

2.	Right	≤ 6 mm	2.30 ± 0.50^B^	3.1 ± 0.31	1.85 ± 0.39^B^	2.50 ± 0.37
		6.01–7.99 mm	0.23 ± 0.12	0.31 ± 0.13	0.25 ± 0.10	0.05 ± 0.05
		≥ 8.00 mm	0.31 ± 0.13	0.23 ± 0.12	0.65 ± 0.11	0.30 ± 0.10
		Overall	2.85 ± 0.45	3.69 ± 0.35	2.75 ± 0.32^B^	2.85 ± 0.33

3.	Overall	≤ 6 mm	4.75 ± 0.86	4.69 ± 0.67	5.55 ± 0.54	5.20 ± 0.44
		6.01–7.99 mm	0.50 ± 0.13	0.38 ± 0.15	0.50 ± 0.17	0.10 ± 0.07
		≥ 8.00 mm	0.25 ± 0.11	0.75 ± 0.14	0.90 ± 0.10	0.85 ± 0.11
		Overall	6.77 ± 0.75	7.15 ± 0.49	6.95 ± 0.47	6.15 ± 0.44

*Note:* Superscripts a, b represent significant different values within rows (*p* < 0.05). Superscripts A, B represent significant different values within columns (*p* < 0.05).

**FIGURE 4:**
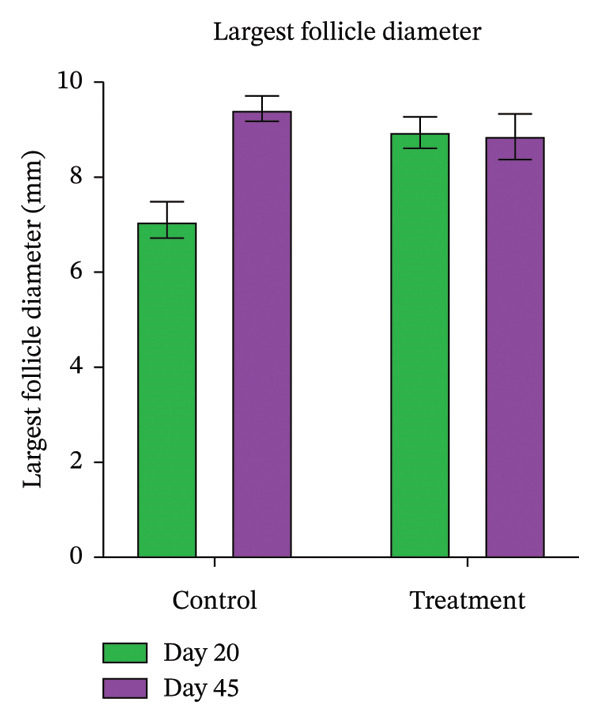
The bar diagram depicting diameter (mm) of the largest ovarian follicle on Days 20 and 45 as examined by ultrasonography in control and treatment groups.

**FIGURE 5:**
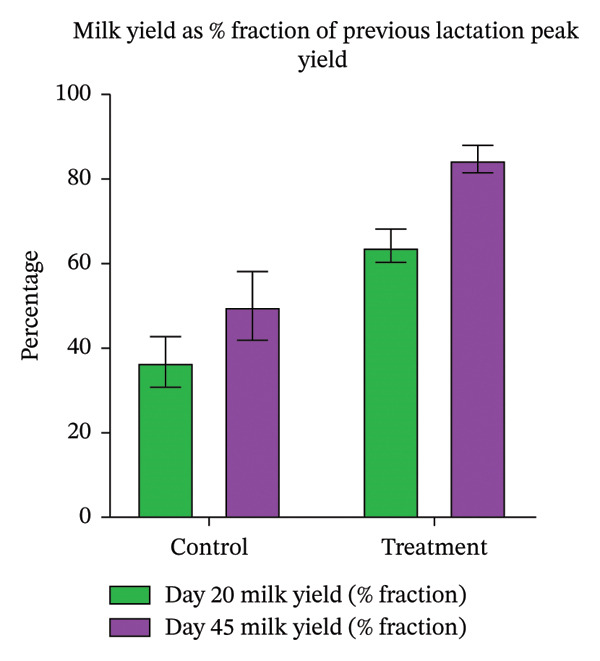
The bar diagram depicting milk yield as % fraction of the previous lactation peak yield on Days 20 and 45 in control and treatment groups.

Ovarian rebound parameters, including number of follicles (Table 4) and largest follicle diameter (Figure 4), showed no significant correlation with uterine involution. Treated animals had higher (*p* < 0.05) milk yield on both Days 20 and 45 (Table 5), and the percentage of peak yield achieved relative to the previous lactation was greater than in controls (Figure 5).

**TABLE 4:** Postpartum fertility parameters in dystocia‐affected buffaloes of different groups.

S. No.	Parameter	Control (*n* = 15)	Treatment (*n* = 15)	*p*‐value
1.	Number of animals showing spontaneous estrus (%)	86.67 (13/15)	100.00 (15/15)	0.483
2.	Calving to first postpartum estrus interval (days)	54.15 ± 3.89	44.80 ± 3.06	0.067
3.	Interval from calving to first AI (days)	80.64 ± 8.70	74.53 ± 5.95	0.554
4.	Service period (days)/open days	129.7 ± 15.07	117.00 ± 15.64	0.597
5.	Conception rate (%) till 120 days postpartum	20.00 (3/15)	66.67 (10/15)	0.025
6.	Overall pregnancy rate (%) till 250 days postpartum	46.67 (7/15)	86.67 (13/15)	0.050

**TABLE 5:** Milk yield (kg) of dystocia‐affected buffaloes in current and previous lactations.

S. No.	Group	Day 20		Day 45		Previous lactation peak milk yield (kg)
		Milk yield (kg)	% of previous lactation peak yield	Milk yield (kg)	% of previous lactation peak yield	
1.	Control (*n* = 15)	5.50 ± 0.58^aA^	36.71 ± 5.89^A^	7.65 ± 0.91^aA^	50.09 ± 8.12^A^	12.54 ± 0.72^b^
2.	Treatment (*n* = 15)	8.48 ± 0.89^aB^	64.08 ± 3.80^B^	10.98 ± 0.80^bB^	84.66 ± 3.25^B^	12.97 ± 0.67^b^

*Note:* Mean ± SEM with superscripts a and b are statistically significant (*p* < 0.05) within rows. Mean ± SEM with superscripts A and B are statistically significant (*p* < 0.05) within columns.

Correlation analysis (Table 6) demonstrated significant positive associations (*p* < 0.001) between uterine involution score and milk yield, as well as cervix, uterine body, and horn diameters. Biochemically, bLac and CK levels decreased significantly (*p* < 0.05) by Days 20 and 45 in treated animals compared with Day 0, and bLac remained significantly (*p* < 0.05) lower than in controls on both days, while CK was significantly lower only on Day 45 (Table 7). AST was significantly lower (*p* < 0.05) in treated animals on Day 20, with other biochemical indices (TP, BUN, and creatinine) unaffected. Across groups, higher bLac and CK levels (*p* < 0.05) were associated with any grade of uterine infection (Table 8). bLac correlated strongly negatively (*p* < 0.05) and CK moderately negatively with uterine involution; AST had a weak positive correlation, and TP, BUN, and creatinine showed no association (Table 9).

**TABLE 6:** Pearson’s correlation (*r*) of uterine involution score with various other parameters irrespective of treatment.

Parameter	“*r*” value in relation to uterine involution score	95% confidence interval	*R* ^2^ (coefficient of determination)	*p*‐value
Milk production (kg)	0.4606^∗^	0.2200 to 0.6484	0.2122	< 0.001
Neutrophils (%)	−0.6396^∗^	−0.7747 to −0.4487	0.4091	< 0.001
No. of follicles	−0.1210	−0.3765 to 0.1517	0.01463	0.384
Largest follicle diameter (mm)	0.2157	−0.05522 to 0.4571	0.04654	0.117
Cervix diameter (mm)	−0.8584^∗^	−0.9157 to −0.7669	0.7368	< 0.001
Uterine body diameter (mm)	−0.8554^∗^	−0.9139 to −0.7622	0.7317	< 0.001
Gravid horn diameter (mm)	−0.8318^∗^	−0.8993 to −0.7256	0.6918	< 0.001
Nongravid horn diameter (mm)	−0.8536^∗^	−0.9127 to −0.7594	0.7286	< 0.001

^∗^Values are significant.

**TABLE 7:** Blood biochemical parameters in control and treatment groups.

S. No.	Parameter	Control (*n* = 15)			Treatment (*n* = 15)		
		Day 0	Day 20	Day 45	Day 0	Day 20	Day 45
1.	bLac	7.78 ± 0.74^ab^	7.89 ± 1.20^abA^	7.25 ± 0.95^A^	7.95 ± 0.74^a^	4.91 ± 0.53^bcB^	4.24 ± 0.39^cB^
2.	CK	543.3 ± 95.27	313.5 ± 83.81	298.0 ± 62.38^caA^	556.3 ± 80.51^a^	263.4 ± 42.29^bc^	143.1 ± 10.79^bB^
3.	AST	183.9 ± 19.02	196.7 ± 27.26^A^	168.3 ± 19.19	192.4 ± 21.33	169.7 ± 11.23^B^	167.2 ± 11.17
4.	Total protein	7.25 ± 0.68	6.96 ± 0.87	7.52 ± 0.56	6.94 ± 0.48	8.30 ± 0.50	9.30 ± 0.68
5.	BUN	13.90 ± 2.33	14.20 ± 1.32	16.10 ± 1.79	20.30 ± 2.20	17.20 ± 1.98	18.90 ± 1.75
6.	Creatinine	1.62 ± 0.19	1.53 ± 0.13	1.68 ± 0.19	1.87 ± 0.18	1.47 ± 0.11	1.65 ± 0.15

*Note:* Mean ± SEM with superscripts a, b, and c are statistically significant (*p* < 0.05) within the same group on different days. Mean ± SEM with superscripts A, B, and C are statistically significant (*p* < 0.05) between two groups on the same corresponding day. Mean ± SEM without superscripts were statistically nonsignificant (*p* > 0.05).

Abbreviations: AST = aspartate aminotransferase, bLac = blood lactate, BUN = blood urea nitrogen, CK = creatine phosphokinase/kinase.

**TABLE 8:** Blood biochemical parameters with respect to enzyme therapy and uterine infections on Days 20 and 45 postpartum in dystocia‐affected buffaloes.

S. No.	Parameters	Day 20 postpartum		Day 45 postpartum	
		Animals developing puerperal metritis (*n* = 15)	Animals not developing puerperal metritis (*n* = 15)	Animals developing clinical and subclinical endometritis (*n* = 9)	Animals not developing clinical and subclinical endometritis (*n* = 21)
1.	bLac	9.12 ± 0.78^aA^	4.35 ± 0.73^B^	6.12 ± 0.89^bA^	3.48 ± 0.65^B^
2.	CK	724.12 ± 65.12^aA^	312.15 ± 33.12^B^	489.18 ± 35.65^bA^	168.12 ± 28.37^B^
3.	AST	212.78 ± 12.32	184.31 ± 23.12	194.21 ± 30.23	169.23 ± 29.16
4.	Total protein	7.89 ± 0.24	7.16 ± 0.34	6.96 ± 0.87	7.46 ± 0.57
5.	BUN	14.70 ± 1.22	17.20 ± 1.81	13.56 ± 1.32	14.20 ± 1.32
6.	Creatinine	1.57 ± 0.13	1.63 ± 0.17	1.61 ± 0.14	1.48 ± 0.18

*Note:* Mean ± SEM with superscripts a, b, and c are statistically significant (*p* < 0.05) within same group on different days. Mean ± SEM with superscripts A, B, and C are statistically significant (*p* < 0.05) between two groups on the same corresponding day. Mean ± SEM without superscripts were statistically nonsignificant (*p* > 0.05).

Abbreviations: AST = aspartate aminotransferase, bLac = blood lactate, BUN = blood urea nitrogen, CK = creatine phosphokinase/kinase.

**TABLE 9:** Correlation of various bold biochemical parameters with uterine involution on both Days 20 and 45 postpartum.

Parameter	UI	blac	CK	AST	C	UB	GUH
blac	−0.779^∗^						
CK	−0.426^∗^	0.113					
AST	0.236	−0.240	−0.321^∗^				
C	−0.768^∗^	0.483^∗^	0.149	−0.033			
UB	−0.862^∗^	0.662^∗^	0.556^∗^	−0.248	0.763^∗^		
GUH	−0.570^∗^	0.424^∗^	0.527^∗^	0.079	0.439^∗^	0.605^∗^	
NGUH	−0.542^∗^	0.349^∗^	0.397^∗^	−0.151	0.693^∗^	0.748^∗^	0.725^∗^

Abbreviations: AST = aspartate aminotransferase, blac = blood lactate, C = diameter of cervix, CK = creatine kinase, GUH = diameter of gravid uterine horn, NGUH = diameter of nongravid uterine horn, UB = diameter of uterine body, UI = uterine involution.

^∗^indicates significant correlation (*p* < 0.05).

The pregnancy rate until Day 250 postpartum was numerically higher in treated buffaloes (80% vs. 46.67%), with descriptive analysis indicating that enzyme therapy was beneficial for improvement in pregnancy outcome but that effect did not reached statistically significant (*p* = 0.067). The factors like uterine involution score at Day 45 (*p* = 0.02), neutrophil count at Day 45 (*p* = 0.004), and uterine infection rates at Days 20 (*p* = 0.034) and 45 (*p* = 0.001) affected pregnancy outcomes individually in a significant manner. However, the binary composite logistic regression pf all these factors on pregnancy outcome showed no combined effect (*p* > 0.05) which might be due to a small sample size.

## 4. Discussion

In dystocia‐affected buffaloes, the normal process of uterine involution is commonly prolonged by approximately 5–14 days beyond the typical postpartum timeline [3]. This delay is not simply a result of physical stress during parturition but is strongly influenced by the higher likelihood of postpartum uterine infections. Such infections are especially common following manual or instrumental handling during dystocia and directly interfere with the normal repair and remodeling of the uterus and cervix [3, 29, 30].

The present study confirmed this pattern. During the first 20 days postpartum, cervical involution proceeded more rapidly than uterine involution, which can be attributed to the smaller mass of cervical tissue and its relatively simpler structural reorganization requirements. After this early phase, the process reversed: The uterus began to involute faster than the cervix. This shift is consistent with the healing sequence previously reported [31, 32], where uterine myometrial contractility improves in the later postpartum period, allowing faster size reduction than that seen in the cervix. Ultimately, complete cervical involution, irrespective of whether the buffalo experienced eutocia or dystocia, was observed by Day 45 postpartum, in agreement with earlier findings [33].

Importantly, buffaloes treated with proteolytic enzymes demonstrated greater reduction in cervical diameter at both Day 20 and Day 45 postpartum compared with untreated controls. This difference suggests a dual mode of action for proteolytic enzymes: first, a biochemical effect as “biological scalpels” that enzymatically break down necrotic tissue and fibrin adhesions, allowing debris to be more readily expelled; and second, a physiological effect by enhancing myometrial contractions, which accelerates the expulsion of lochia and bacterial contaminants. This early and effective clearance of postpartum uterine contents reduces the microbial load, thereby limiting the severity and duration of infection, which in turn supports faster tissue repair.

The earlier reduction in the size of both uterine horns and the uterine body in treated animals may also be linked to protease‐mediated stimulation of endometrial repair processes. This includes faster epithelial regeneration, reduced edema, and improved vascular perfusion in the uterine wall. Furthermore, the treatment likely amplified the chemotactic response of neutrophils, which are critical for bacterial clearance and tissue debridement, leading to a shortened inflammatory phase and earlier transition to tissue remodeling [34].

By contrast, in the control group, a higher incidence of retained fetal membranes and uterine infections was observed. These complications are expected after dystocia because of the mechanical manipulation during delivery, increased trauma to the endometrium, and the favorable conditions created for pyogenic bacterial colonization [30, 35, 36]. The proteolytic enzyme‐treated animals benefited from early fetal membrane detachment, likely due to enzymatic hydrolysis of peptide bonds at the cotyledon–caruncle interface, reducing the time the membranes remained attached and thereby minimizing the risk of bacterial invasion, as previously reported by this laboratory [34].

The chymotrypsin component of the enzyme preparation may have further contributed by directly stimulating smooth muscle contractility [15], thereby aiding both uterine clearance and cervical closure. These physiological and biochemical effects, acting in synergy, explain the accelerated uterine involution observed in the treatment group. Such enzyme‐mediated benefits have already been documented for infection clearance and fertility improvement in both cattle [17, 19] and buffaloes [18, 20]. The modulation of pro‐ and anti‐inflammatory cytokines reported for proteolytic enzymes [18] likely played a role here as well, allowing a more balanced inflammatory response that favored tissue healing over prolonged immune activation.

In the early postpartum phase (Day 20), the contralateral ovary to the gravid uterine horn contained more follicles than the ipsilateral ovary. This observation supports the concept that the resumption of ovarian cyclicity often begins in the ovary opposite to the pregnant horn [37, 38]. The likely explanation lies in localized uterine–ovarian signaling: The uterus adjacent to the ipsilateral ovary may still be undergoing significant tissue repair and involution, potentially influencing the ipsilateral ovary via vascular or prostaglandin‐mediated pathways.

Contrary to the earlier work of Agrawal et al. [39], which reported a predominance of small follicles during the early puerperium, the present study found similar total follicle counts in both treatment and control groups at both Day 20 and Day 45. This suggests that in these dystocia‐affected buffaloes, overall follicular recruitment was not impaired by the postpartum condition or the treatment. Moreover, ovarian activity appeared largely independent of uterine involution, in line with the findings of Sheldon et al. [28], indicating that the hypothalamic–pituitary–ovarian axis can recover and resume follicular activity regardless of uterine status, provided systemic illness is not severe.

However, the size of the largest follicle differed between groups. In the control group, the largest follicle averaged less than 8 mm on Day 20, consistent with the expected slower progression toward ovulation [40]. In the treatment group, however, the mean largest follicle size was 8.95 ± 0.34 mm at the same time point, indicating that follicular growth was more advanced, potentially allowing ovulation to occur earlier in the postpartum period. This difference may be linked to reduced uterine infection rates in treated animals, as infections are known to release LPS, which can directly suppress granulosa cell aromatase activity, lower estradiol production, and delay ovulation [41].

Furthermore, LPS may impair GnRH and LH secretion from the hypothalamus and pituitary, disrupting LH pulsatility and thereby preventing the LH surge necessary for ovulation [42]. By reducing uterine infections, proteolytic enzyme treatment may have preserved the normal endocrine environment needed for timely ovulation. By Day 45, follicle sizes were comparable between groups, suggesting that the initial advantage in follicular development in treated animals primarily influenced the early postpartum phase rather than long‐term follicular growth.

Postpartum fertility in dystocia‐affected buffaloes was lower than in animals with normal calving histories, as evidenced by a longer service period compared to their previous lactation and lower reproductive efficiency overall. In their previous lactations, these same animals had shorter service periods and higher milk yields, indicating that dystocia had a negative carryover effect on reproductive and productive performance.

Proteolytic enzyme treatment appears to have mitigated some of this impact. By promoting rapid uterine epithelial restoration and ensuring early expulsion of fetal membranes, treated animals achieved earlier return to reproductive normalcy and higher pregnancy rates. The improved uterine environment likely supported the survival of early embryos and reduced embryonic loss. Although several individual variables showed significant associations with pregnancy outcome, the multivariable binary logistic regression analysis in the study revealed a nonsignificant combined effect of different variables on pregnancy outcome; it should be interpreted cautiously. This might be due to the relatively limited number of animals included in the study, which may result in the reduced statistical power of the model to detect combined effects. The absence of statistical significant results in the regression analysis should not be interpreted as the absence of biological influence, and further studies with larger sample sizes to establish these relationships are recommended.

In terms of milk production, the treatment group outperformed the control group on both Day 20 and Day 45 postpartum. This can be explained by a lower burden of systemic illness in treated animals, reflected in fewer infections, lower neutrophil counts, and maintenance of normal feeding behavior. Healthy postpartum physiology allows for better nutrient partitioning toward lactation rather than immune system demands. However, the milk yields in both groups were lower than in the animals’ previous lactations, aligning with earlier findings that dystocia reduces both daily and total lactation output [43]. Severe dystocia in particular has been shown to markedly impair milk yield [44], while mild dystocia exerts minimal effects; similar trends have been reported in Holstein cows [45].

bLac is a reliable indicator of anaerobic metabolism and rises in response to poor tissue perfusion, venous congestion, or cellular injury. Elevated bLac in cases of uterine torsion has been linked to uterine necrosis and poor prognosis [22, 23]. In this study, treated animals had significantly lower bLac levels at both Day 20 and Day 45 postpartum compared with controls. This suggests that proteolytic enzyme therapy not only promoted faster involution but also improved tissue oxygenation and reduced anaerobic metabolism in the uterine environment.

The CK levels, which reflect muscle cell damage, were significantly reduced over time in treated animals and were lower than controls by Day 45. Given that CK originates from muscular trauma during difficult deliveries and uterine manipulation, these reductions indicate faster resolution of tissue injury. Previous studies in both bovines and buffaloes have linked high CK levels to prolonged dystocia, severe inflammation, and poor prognosis [23, 24]. In the present study, reduced CK levels coincided with clinical evidence of improved uterine health, reinforcing its potential as a prognostic marker.

It is emphasized that serum biochemical markers such as bLac and CK are not only associated with uterine recovery but also the degree of stress associated with dystocia and obstetrical manipulation. Various factors like the duration of labor, the severity of uterine torsion, the duration of handling of obstetrical cases, and the extent of manual intervention can influence these biomarkers. Therefore, in the current study, efforts were made to minimize this potential confounding effect. The types of animals in the treatment and control groups with respect to the type and cause of dystocia were rationalized and were equally distributed as detailed in the supporting tables. So, it is recommended that the contribution of dystocia severity to these biochemical alterations should be considered while interpreting these markers in clinical settings.

AST varied nonsignificantly across time points, though control animals had higher levels on Day 20, possibly due to higher infection prevalence. Elevated AST in previous studies has been associated with ischemic muscle injury during dystocia and with stress‐induced glucocorticoid release [46]. The lower AST values in treated animals by Day 45 may reflect reduced muscle stress and complete involution.

TP levels did not differ significantly between groups but were slightly higher in treated animals on Day 20. This suggests better feed intake and protein metabolism in these animals, as TP reflects both hepatic protein synthesis and nutritional status. In control animals, reduced TP may have been due to decreased feed intake, diversion of proteins toward inflammatory processes, and leakage into inflamed uterine tissues [47].

Both BUN and creatinine remained within normal ranges in all animals. Unlike earlier studies, no significant elevations were observed, likely because sampling here extended into later postpartum stages, by which time acute kidney stress due to dystocia‐related shock or fetal decomposition would have subsided.

The authors acknowledge certain limitations in the study despite the promising findings. The sample size in the current study was relatively small and limited to only dystocia‐affected buffaloes presented at a single clinical facility. Due to this smaller sample size, the statistical power of the study is weaker, although strong biological variations were identified. The statistical results of pregnancy analysis as well as composite regression analysis were not significant, and they must be interpreted cautiously. Therefore, it is warranted that the study must be implemented on a large scale for generalization of the results to broader buffalo populations. In addition, other environmental and management factors affecting the process of parturition and leading to difficult birth were not included in the study, as it was conducted at the referral veterinary clinics, not at the dairy farm or in field conditions.

It should be noted that all animals in both groups received conventional postpartum therapy, including antimicrobials, NSAIDs, ecbolics, and supportive treatment as part of routine clinical management. Therefore, the beneficial effects observed in the treatment group due to intrauterine proteolytic enzyme therapy should be interpreted as additional advantages of this therapy when administered alongside standard postpartum care, rather than as an independent replacement for conventional therapeutic approaches.

## 5. Conclusions

The intrauterine proteolytic enzyme therapy consisting of trypsin, chymotrypsin, and papain in addition to conventional postpartum therapeutic management resulted in early uterine involution, reduced uterine infections, as well as timely resumption of ovarian cyclicity and conception in dystocia‐affected buffaloes. It indicates the additive beneficial effects of this therapy, which should not be interpreted as the effect of this therapy alone. In addition, this therapy was also beneficial in terms of improvement in milk production, where treated animals achieved approximately 85% of the milk yield recorded in their previous normal lactation, which was higher than that observed in control animals. Further, bLac and CK could be considered as potential biomarkers of uterine health. However, a more comprehensive study is warranted in animals with normal and difficult calving to promote the therapy as an alternative approach to antimicrobials against uterine infections in dairy animals.

## Author Contributions

Nakul Gulia: conducted the experiment and writing of the manuscript. Mrigank Honparkhe: design of study, overall supervision, manuscript correction, and proofreading. Amarjeet Bisla: experiment conducting, manuscript writing, and data statistical analysis. Ashwani K. Singh: field data collection, statistical analysis. Prahlad Singh: design of the study, manuscript correction, and proofreading.

## Funding

This work was supported by Indian Council of Agricultural Research under the research project entitled “Nutritional and Physiological Approaches for Enhancing Reproductive Performance in Cattle and Buffalo” (AICRP; ICAR‐19).

## Disclosure

All authors read and gave final approval to the manuscript before submission.

## Conflicts of Interest

The authors declare no conflicts of interest.

## Supporting Information

Additional supporting information can be found online in the Supporting Information section.

## Supporting information


**Supporting Information** The supporting file consists of three tables describing the types of uterine torsion in animals included in both groups (Supporting Table 1), type of obstetrical procedures used to correct dystocia (Supporting Table 2) and the conventional medicinal therapy given to all animals (Supporting Table 3).

## Data Availability

All data generated during the study is included within the manuscript.
