# Supplementary material for: Impact of Postcalving Intrauterine Enzymes Therapy on Uterine Health, Fertility, Biochemical Indices, and Milk Production in Dystocia‐Affected Buffaloes
Source: Vet Med Int. 2026 May 12;2026:5748390. doi: 10.1155/vmi/5748390 (PMC13162126; doi:10.1155/vmi/5748390)
Supplement: Supplementary file 1 — Supporting Information The supporting file consists of three tables describing the types of uterine torsion in animals included in both groups (Supporting Table 1), type of obstetrical procedures used to correct dystocia (Supporting Table 2) and the conventional medicinal therapy given to all animals (Supporting Table 3). [file VMI-2026-5748390-s001.docx]

**Supplementary Data**

**Supp Table 1. Various types of uterine torsion in buffaloes**

| **S. No.** | **Groups** | | **Based on location** | | **Based on side** | | **Based on degree of rotation** | |
| --- | --- | --- | --- | --- | --- | --- | --- | --- |
|  |  |  | **Pre-cervical** | **Post-cervical** | **Right** | **Left** | **<180** | **180-270** |
| 1. | Control (n=7) | No. of animals | 02 | 05 | 07 | - | 03 | 04 |
|  |  | % | 28.57 | 71.43 | 100 | - | 42.85 | 57.15 |
| 2. | Treatment (n=8) | No. of animals | 01 | 07 | 07 | 01 | 03 | 05 |
|  |  | % | 12.50 | 87.50 | 87.50 | 12.50 | 37.50 | 62.50 |
| 3. | Overall (n=15) | No. of animals | 03 | 13 | 14 | 01 | 06 | 09 |
|  |  | % | 20.00 | 80.00 | 93.33 | 6.67 | 40.00 | 60.00 |

**Supp Table 2. Various obstetrical operations applied to correct dystocia in buffaloes**

| **S. No.** | **Obstetrical operation** | **Control (n=15)** | | **Treatment (n=15)** | | **Overall (n=30)** | |
| --- | --- | --- | --- | --- | --- | --- | --- |
|  |  | **No. of animals** | **Percentage** | **No. of animals** | **Percentage** | **No. of animals** | **Percentage** |
| 1. | Mutation and Traction | 10 | 66.67 | 11 | 73.33 | 21 | 70.00 |
| 2. | Fetotomy | 05 | 33.33 | 04 | 26.67 | 09 | 30.00 |

**Supp Table 3. Conventional medicinal therapy given to all dystociac animals**

| **S. No.** | **Class of medication** | **Name of drug** | **Dosage** | **Brand and Manufacturer’ name** |
| --- | --- | --- | --- | --- |
|  | IV fluid | Normal Saline  Ringer’s lactate | @ 10 mL/kg b.wt.  @ 10 mL/kg b.wt. |  |
|  | Calcium Therapy | Calcium Magnesium borogluconate | 450 mL IV (slow) | Mifex, Elanco |
|  | Antimicrobials | Ceftiofur | @ 2.2. mg/kg b.wt. OD IM | Xceft, Alembic |
|  |  | Metronidazole | @ 20 mg/kg b.wt. OD IV | Merogyl, |
|  | NSAIDs | Flunixine meglumine | @ 2.2 mg/kg b.wt. OD IM | Megludyne, Virbac |
|  | Multivitamins | --- | 10 mL OD IM | Tribivet, Neovet |
|  | Liver Extract | -- | 10 mL OD IM | Belamyl, Zydus |
|  | Ecbolics | Ergometrine | 10 mL OD IM | Ergovet, Carus labs |
|  |  | Oral Uterine cleanser | 200 mL PO BD | Involon, natural remedies |
|  | Rumenotorics | --- |  | Rumentas, Intas Pharmaceuticals |
|  | Antioxidants | Vitamin E and Selenium | 10 mL OD SC | Repronol, Cadilla |

IV; Intravenous, OD; Once a day, IM; Intramuscular, PO; Orally, BD; Twice daily, SC; Subcutaneous, NSAIDs; Non-steroidal anti-inflammatory drugs
